# A phase Ib/II study of eribulin in combination with cyclophosphamide in patients with advanced breast cancer

**DOI:** 10.1007/s10549-023-07073-0

**Published:** 2023-10-10

**Authors:** Ozge Gumusay, Laura A. Huppert, Mark Jesus M. Magbanua, Chiara A. Wabl, Michael Assefa, Amy Jo Chien, Michelle E. Melisko, Melanie C. Majure, Mark Moasser, John Park, Hope S. Rugo

**Affiliations:** 1 Department of Medical Oncology, School of Medicine, Acibadem University, Istanbul, Turkey; 2 University of California San Francisco, Helen Diller Family Comprehensive Cancer Center, San Francisco, CA USA; 3 Department of Laboratory Medicine, UCSF, San Francisco, CA USA; 4 Atlanta, GA USA

**Keywords:** Eribulin, Cyclophosphamide, Metastatic breast cancer, Chemotherapy

## Abstract

**Purpose:**

We hypothesized that eribulin combined with cyclophosphamide (EC) would be an effective combination with tolerable toxicity for the treatment of advanced breast cancer (ABC).

**Methods:**

Patients with histologically confirmed metastatic or unresectable ABC with any number of prior lines of therapy were eligible to enroll. In the dose escalation cohort, dose level 0 was defined as eribulin 1.1 mg/m^2^ and cyclophosphamide 600 mg/m^2^_,_ and dose level 1 was defined as eribulin 1.4 mg/m^2^ and cyclophosphamide 600 mg/m^2^. Eribulin was given on days 1 and 8 and cyclophosphamide on day 1 of a 21-day cycle. In the dose expansion cohort, enrollment was expanded at dose level 1. The primary objective was clinical benefit rate (CBR), and secondary objectives were response rate (RR), duration of response (DOR), progression-free survival (PFS), and safety.

**Results:**

No dose-limiting toxicities were identified in the dose escalation cohort (n = 6). In the dose expansion cohort, an additional 38 patients were enrolled for a total of 44 patients, including 31 patients (70.4%) with hormone receptor-positive (HR +)/HER2- disease, 12 patients (27.3%) with triple-negative breast cancer (TNBC), and 1 patient (2.3%) with HR + /HER2 + disease. Patients had a median age of 56 years (range 33–82 years), 1 prior line of hormone therapy (range 0–6), and 2 prior lines of chemotherapy (range 0–7). CBR was 79.5% (35/44; 7 partial response, 28 stable disease) and the median DOR was 16.4 weeks (range 13.8–21.1 weeks). Median PFS was 16.4 weeks (95% CI: 13.8–21.1 weeks). The most common grade 3/4 adverse event was neutropenia (47.7%, n = 21). Fourteen of 26 patients (53.8%) with circulating tumor cell (CTC) data were CTC-positive ($\ge$ 5 CTC/7.5 mL) at baseline. Median PFS was shorter in patients who were CTC-positive vs. negative (13.1 vs 30.6 weeks, p = 0.011).

**Conclusion:**

In heavily pretreated patients with ABC, treatment with EC resulted in an encouraging CBR of 79.5% and PFS of 16.4 weeks, which compares favorably to single-agent eribulin. Dose reduction and delays were primarily due to neutropenia. The contribution of cyclophosphamide to eribulin remains unclear but warrants further evaluation. NCT01554371.

**Supplementary Information:**

The online version contains supplementary material available at 10.1007/s10549-023-07073-0.

## Introduction

Breast cancer is the most common malignancy affecting women worldwide [1]. In the United States, approximately 5% of patients with breast cancer have de novo metastatic disease at diagnosis, and at least 20% of those initially diagnosed with early-stage breast cancer subsequently develop metastatic disease [2]. Despite advances in therapies, the prognosis of patients with metastatic breast cancer (MBC) remains poor, with a median time to progression on first-line therapy of 9.7 months for triple-negative breast cancer (TNBC) and 25.3 months for HR + , HER2 negative breast cancer and a median overall survival (OS) of 23 months for TNBC and 63.9 months for HR + , HER2 negative breast cancer [3–5]. Patients with metastatic disease develop cumulative toxicity from multiple lines of chemotherapy, as well as chemotherapy resistance, which limits efficacy. In order to improve survival while maintaining quality of life, it is important to identify new treatment regimens for patients with MBC. New chemotherapy combinations may improve the duration of response (DOR) and also may provide a superior chemotherapy backbone for the addition of targeted agents in future studies.

Eribulin mesylate, a nontaxane microtubule dynamics inhibitor, is a structurally simplified, synthetic analog of the natural product Halichondrin B, isolated from the marine sponge *Halichondria okadai* [6, 7]. Eribulin suppresses polymerization and sequesters tubulin into nonfunctional aggregates [6, 8–10] and has other cytotoxic effects, including vascular remodeling, reversal of the epithelial–mesenchymal transition, induction of the differentiation, and suppression of migration and invasion [11, 12]. Eribulin is administered as a 2- to 5-min IV infusion without the need for premedications, so it is easier to administer than other chemotherapy agents [13]. Eribulin was approved as monotherapy for the treatment of taxane and anthracycline-resistant MBC based on results from the EMBRACE trial, which reported a 2.5-month improvement in median OS with eribulin compared to treatment of physicians choice (TPC, 13.1 months vs 10.6 months; HR 0.81, 95% CI 0.66–0.99 p = 0.041) in women with heavily pre-treated MBC [14]. Response rate (RR) was significantly longer with eribulin, with a non-significant numerical difference in PFS. In another study, eribulin was compared to capecitabine as an earlier line therapy (up to two lines of chemotherapy) and showed no difference in OS or PFS, but a pooled subset analysis suggested improved OS with eribulin in patients with HER2-negative and TNBC [15, 16]. In both studies, treatment-related adverse events (AEs) with eribulin included neuropathy and neutropenia, requiring higher rates of growth factor administration [17–19].

Combination chemotherapy with docetaxel and cyclophosphamide (TC) has become a standard treatment option for early-stage lower risk breast cancer based on data from several studies showing improved or similar outcome compared to anthracycline-based regimens [20–22] Compared to doxorubicin and cyclophosphamide (AC), treatment with TC improved OS in patients with up to three positive axillary nodes [20]. Based on encouraging efficacy with TC, we hypothesized that eribulin combined with cyclophosphamide (EC) would be effective in taxane-resistant disease with tolerable toxicity. The aim of this study was to determine the maximum tolerated dose (MTD) of EC, followed by a dose expansion study to estimate the clinical benefit rate (CBR) of EC in patients with advanced breast cancer (ABC). We also performed correlative studies to assess the correlation of circulating tumor cells (CTCs) with response and survival.

## Methods

### Patients

Male or female patients ≥ 18 years who had histologically confirmed locally advanced, unresectable or metastatic carcinoma of the breast of all subtypes were eligible to enroll with no limitation on prior lines of therapy. Eligibility included an Eastern Cooperative Oncology Group performance status (ECOG PS) of 0–2, measurable disease, adequate organ and bone marrow functions (neutrophils > 1.0 × 10^9^/L, platelets > 100 × 10^9^/L, hemoglobin > 9 g/dL, total bilirubin < 1.5 × upper limit of normal (ULN), AST and ALT ≤ 3 × ULN or ≤ 5 × ULN in patients with known liver metastasis, creatinine ≤ 1.5 × ULN or ≥ 60 mL/min for patients with creatinine levels > 1.5 × institutional ULN),  ≤ grade 1 peripheral neuropathy, and a life expectancy of at least 3 months. Patients with stable treated brain metastases were also eligible to enroll. Exclusion criteria included known active central nervous system (CNS) metastases and/or carcinomatous meningitis, a corrected QT interval (cQT) > 480 ms, or significant cardiovascular disease within the past 6 months. The study was approved by the University of California San Francisco (UCSF) Comprehensive Cancer Center Protocol Review Committee on Human Research, and written informed consent was obtained from all patients prior to trial enrollment. The trial was registered at ClinicalTrials.gov (NCT01554371).

### Study design

Patients were treated using a 3 + 3 dose confirmation strategy for eribulin with dose expansion at the MTD. Cyclophosphamide was given at a fıxed dose of 600 mg/m^2^ on day 1 of a 21-day cycle; eribulin was given day 1 and 8 every of a 21 day cycle, and was escalated from 1.1 mg/m^2^ at dose level 0 (DL0) to 1.4 mg/m^2^ at dose level 1 (DL1); both drugs were given intravenously. Dose expansion occurred at DL1. The primary objective of dose escalation was to determine the MTD of EC. The highest dose level at which no more than one of six subjects experienced a dose limiting toxicity (DLT) defined the MTD. DLTs were defined as grade 3 or 4 clinically evident non-hematologic toxicity; grade 4 neutropenia, thrombocytopenia lasting > 7 days, febrile neutropenia or any clinically significant toxicity grade 2 or higher that required more than 14 days to resolve occurring within the first 21 days of combination therapy. The primary objective of the dose expansion was CBR at three months; we selected CBR at 3 months rather than 6 months, as these patients were heavily pre-treated so we anticipated shorter responses to therapy in the later line setting. Secondary objectives were RR, DOR, PFS, safety, and correlation of CTCs with clinical benefit.

Drug doses were modified for treatment-related toxicity. These toxicities were neutropenia (absolute neutrophil count [ANC] < 1000/μL), thrombocytopenia, rash, GI toxicity, liver abnormalities and neuropathy. If toxicity occurred in a patient, dose reductions were managed as follows. Dose levels -1 and -2 for eribulin were 1.1 mg/m^2^ and 0.7 mg/m^2^, for cyclophosphamide one dose reduction was allowed to 500 mg/m^2^. Dose reductions were sequential, with the first dose reduction for cyclophosphamide, followed by eribulin to dose level -1 then -2 for persistent toxicity. If toxicity persisted despite these dose reductions and/or if the participant experienced a cycle delay of three or more weeks, study treatment was discontinued (Supplement 1).

### Concomitant medication

Patients received prophylactic antiemetics and premedications according to standard institutional guidelines. Colony stimulating growth factor use was allowed at the discretion of the treating physician. Palliative radiotherapy was permitted to control bone pain as long as the irradiated area was limited in extent. Other investigational agents and potent inhibitors or inducers of CYP3A4 were not permitted.

### Assessments

Baseline evaluations included medical history, a physical examination, Eastern Cooperative Oncology Group (ECOG) Performance Status (PS) tumor imaging with computed tomography (CT), bone scan, laboratory tests (hematology, blood chemistry), a serum pregnancy test for females of child-bearing potential, and an electrocardiogram with QTc measurement. In the dose confirmation cohort (phase Ib), the response to EC was evaluated every 6 weeks until disease progression according to the investigator, based on objective tumor assessments using RECIST version 1.1 criteria. In the dose-expansion cohort (phase II), response to EC was evaluated after study start, then every 9 weeks until end of study therapy.

### Safety/tolerability

Safety evaluations at baseline and subsequent visits included AEs, clinical laboratory tests, physical examination, and vital signs. AEs were assessed and AE severity was graded in accordance with the National Cancer Institute Common Terminology Criteria for Adverse Events (CTCAE version 4.0). Both agents were held for grade 3 or febrile neutropenia (ANC < 1000/μL). Filgrastim or pegylated-filgrastim myeloid growth factor support was encouraged for ANC < 1500/μL and was allowed at the discretion of the treating physician in order to maintain adequate blood counts. Filgrastim was given for ANC < 1000/μL at any time, or as prophylaxis in patients at risk for neutropenia. Neuropathy was assessed using the 10-point Modified Total Neuropathy Score at the start of each cycle and at study termination.

### Correlative studies

Whole blood samples were obtained in fixative-containing tubes (CellSave tubes, Veridex (currently Menarini)) and processed in the laboratory of Dr. John Park at University of California, San Francisco for CTC identification and enumeration using the CellSearch system. Samples with $\ge$ 5 CTCs per 7.5 mLs of blood were considered CTC-positive.

### Statistical analysis

The study followed a standard dose-confirmation schema (phase Ib portion) with three patients per cohort (3 + 3 design) for a total of six patients. A two-stage design was performed in the dose-expansion (phase II portion) with a possible total of 40 patients. An overall RR of 25% was considered clinically meaningful. Using a two-stage Simon’s minimax design, the null and alternative hypothesis were H0: p0 < 10% versus Ha: p1 > 25% for the proportion of patients with complete or partial response (PR) by RECIST criteria. Based on the type I error of 5% and the type II error rate of 20%, p0 = 10% and p1 = 25%. Secondary efficacy variables were analyzed using Kaplan–Meier methods, with a corresponding median and a 95% CI.

## Results

### Patient characteristics

Patients with histologically confirmed metastatic or ABC with any number of prior lines of therapy were eligible to enroll in this study, and 44 patients enrolled in total. Baseline patient characteristics are summarized in Table 1. The median age was 56 years (range 33–82 years). 31 patients (70.4%) had HR + /HER2- disease, 12 patients (27.3%) had TNBC, and 1 patient (2.3%) had HR + /HER2 + disease. Patients had a median of 1 prior line of hormone therapy (range 0–6) and 2 prior lines of chemotherapy (range 0–7). Most patients (97.7%) had visceral disease. The most common metastatic sites of disease were bone, lymph nodes, liver, and lung (Table 1).

**Table 1:** Patient characteristics at baseline

Characteristics	EC (n = 44)	%
Age, years		
Median (range, yrs)	56 (33–82)	
Sex		
Female	44	100
Male	0	0
Subtype		
HR + /HER2-	31	70.4
TNBC	12	27.3
HR + /HER2 +	1	2.3
Prior lines of chemotherapy		
0	1	2.3
1	15	34.1
2	10	22.7
$\ge$ 3	18	40.9
Prior lines of endocrine therapy		
0–1	24	54.5
2	8	18.2
$\ge$ 3	12	27.3
Metastatic disease		
Bone only disease	1	2.3
Visceral disease	8	18.2
Bone and visceral disease	35	79.5
Metastatic sites		
Bone	34	77.3
Lymph node	29	65.9
Liver	29	65.9
Lung	20	45.5
Soft tissue involvement	8	18.2
CNS metastasis	6	13.6
Peritoneum	6	13.6

*CNS* central nervous system, *ECOG* Eastern Cooperative Oncology Group

### Antitumor activity

**Fig. 1:**
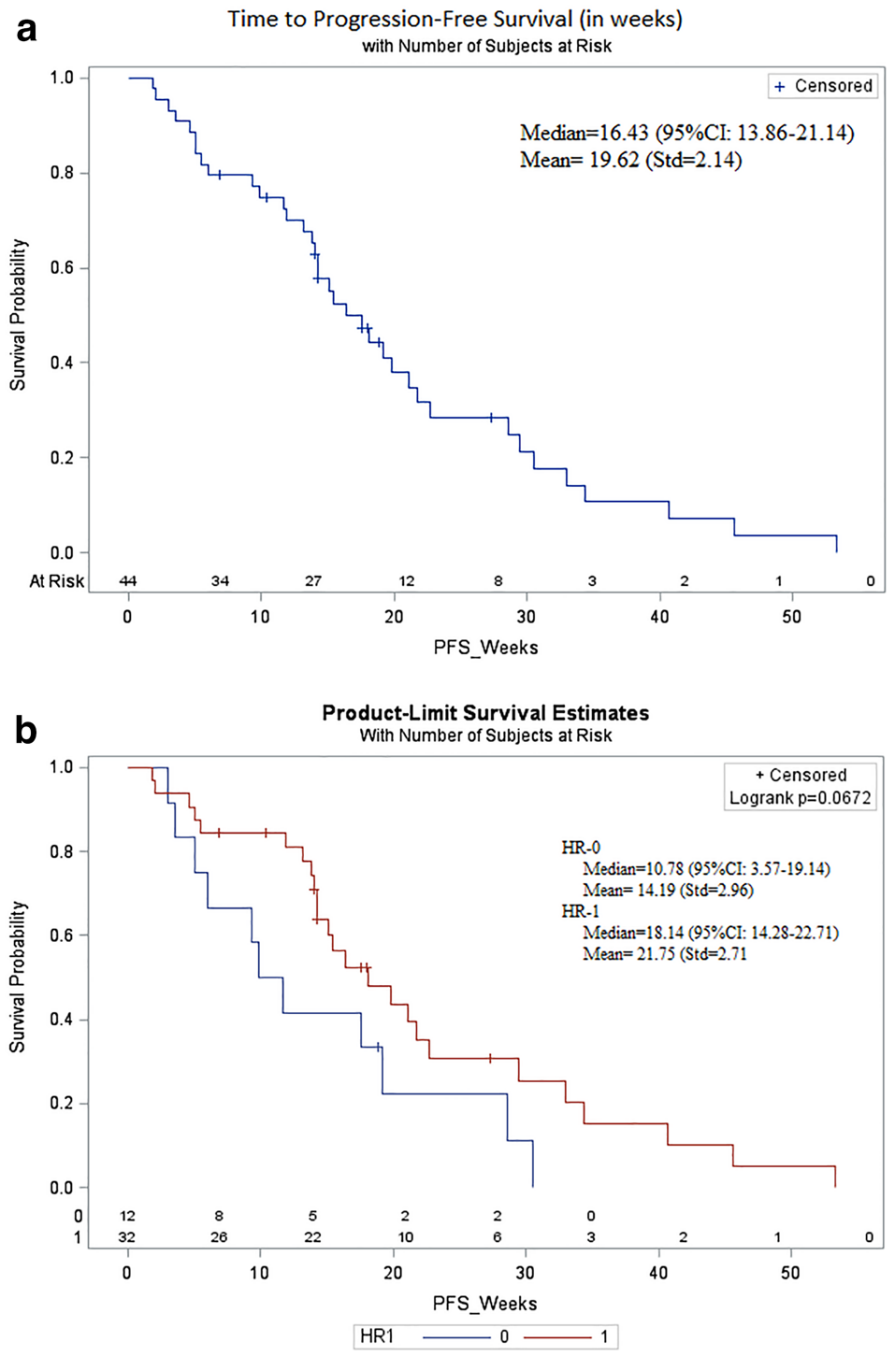
Kaplan–Meier plot of progression-free survival (PFS). **a** Shown is the Kaplan–Meier plot for PFS for all patients in the study. The median PFS was 16.4 weeks (95% CI: 13.8–21.1 weeks) in all patients. **b** Shown is the Kaplan–Meier plot for PFS for patients with HR + /HER2- disease (red) and TNBC (blue). The median PFS was 18.1 weeks in patients with HR + /HER2- disease and 10.8 weeks in patients with TNBC

The median duration of treatment was 14.7 weeks (1.8–53.3 weeks). The CBR was 79.5% (35/44; 7 PR, 28 SD). The median PFS was 16.4 weeks (95%CI: 13.8–21.1 weeks). (Figure 1a). The median DOR was 16.4 weeks (13.8–21.1 weeks). Clinical response to EC therapy is summarized in Table 2. Individual patient characteristics of those who had a PFS $\ge$ 24 weeks on EC are summarized in Table 3.

**Table 2:** Clinical response to EC therapy

Response endpoints	EC (N = 44)
CBR	35 (79.5)
CR, n (%)	0 (0)
PR, n (%)	7 (15.9)
SD, n (%)	28 (63.6)
PD, n (%)	6 (13.6)
n/a, n (%)	3 (6.8)
CBR (CR + PR + SD $\ge$ 6 months), n (%)	9 (20.5)
PFS, weeks (median) 95% CI	16.4 (13.8–21.1)
PFS, weeks for HR + vs TNBC patients	18.1 vs 10.8 (p = 0.067)

*CBR* clinical benefit rate, *CI* confidence interval, *CR* complete response, *n* number, *PD* progressive disease, *PR* partial response, *PFS*, progression-free survival, *SD* stable disease

**Table 3:** Individual patient characteristics whose PFS with EC $\ge$ 24 weeks

Patient no	Ph	Subtype	Prior lines of ET	Prior lines of CT	Metastatic sites	PFS (weeks)
101	Ib	HR + /HER2-	3	3	Bone only	27.3
103	Ib	HR + /HER2-	0	1	Bone and visceral	40.7
107	Ib	HR + /HER2-	2	1	Bone and visceral	34.4
208	II	TNBC	0	3	Bone and visceral	30.6
213	II	HR + /HER2-	3	2	Bone and visceral	33.0
219	II	HR + /HER2-	1	3	Bone and visceral	53.3
228	II	HR + /HER2-	1	2	Bone and visceral	45.0
235	II	TNBC	0	2	Bone and visceral	28.6
240	II	HR + /HER2-	2	2	Bone and visceral	29.4

*CT* chemotherapy, *ET* endocrine therapy, *PFS* progression-free survival

The CBR at 3 months in patients with HR + /HER2- disease was 83.9% (n = 31), and 12.9% of patients had a PR. The median PFS was 18.1 weeks. In the 12 patients with TNBC, the CBR was 66.7% with a PR rate of 25%. The median PFS was 10.8 weeks. As expected, PFS was longer in those with HR + disease versus those with TNBC (18.1 vs 10.8 weeks; p = 0.067) (Fig. 1b).

The CBR among patients who had received (neo)adjuvant treatment with an anthracycline and/or a taxane (A/T) was 81.8% (18/22), similar to that of the overall study population. There was no difference in PFS among patients who had received prior A/T (n = 24) versus those who had treatment without A/T (n = 20) (21.1 vs.15.1 weeks respectively, p = 0.4251). There was no difference in PFS among patients who received 0–2 prior lines of chemotherapy versus who received $\ge 3$ lines of chemotherapy (18.1 vs. 14.7 weeks respectively, p = 0.6736). In patients with HR + disease, patients who had received 0–2 prior lines of chemotherapy (n = 17) had a median PFS of 21.1 weeks whereas patients who had received $\ge 3$ lines of chemotherapy (n = 15) had a median PFS of 14.3 weeks. In patients with TNBC, patients who had received 0–2 prior lines of chemotherapy (n = 9) had a median PFS of 9.8 weeks whereas patients who had received $\ge 3$ lines of chemotherapy (n = 3) had a median PFS of 19.1 weeks.

### Drug exposure and safety

No DLTs were identified in the dose confirmation (phase Ib) portion of the study. Three patients were treated at DL0 (eribulin 1.1 mg/m^2^) and 3 were treated at DL1 (eribulin 1.4 mg/m^2^), the MTD. Thirty-seven patients (84.1%) completed at least three cycles of treatment and 21 (47.7%) received $\ge 6$ cycles of treatment. The median number of cycles delivered was 5.8 (1.1–17.8); the median exposure was 3.6 weeks and nine patients received treatment for 6 months or longer (20.5%). Seventeen patients (38.6%) had $\ge$ 1 cyclophosphamide dose reduction and 12 patients (27.3%) had $\ge$ 1 eribulin dose reduction.

Adverse events (AEs) are summarized in Table 4. Twenty-eight patients (63.6%) experienced a grade 3/4 AE, the most common of which were neutropenia (47.7%, n = 21), fatigue (4.5%, n = 2), dyspnea (4.5%, n = 2), and anemia (2.3%, n = 1). Febrile neutropenia was reported for three patients (6.8%). Most patients (77.3%) received myeloid growth factors. Treatment related AEs led to dose adjustment (interruption/delay or reduction): 26 patients (59.1%) had a dose interruption/delay and 17 patients (38.6%) underwent dose reduction due to a treatment-related AE. Dose reductions due to neutropenia included a decrease of cyclophosphamide to 500 mg/m^2^ (n = 17) and of eribulin to 1.1 mg/m^2^ (n = 12). Five patients discontinued treatment due to fatigue (n = 3) or neutropenia (n = 2).

**Table 4:** Adverse events

Adverse event	Any grade, n (%)	Grade 1–2, n (%)	Grade 3–4, n (%)
Fatigue	30 (68.2)	28 (63.6)	2 (4.5)
Neutrophil count decrease	26 (59.1)	5 (11.4)	21 (47.7)
Nausea	25 (56.8)	25 (56.8)	0 (0)
Constipation	22 (50)	22 (50)	0 (0)
Peripheral neuropathy	21 (47.7)	21 (47.7)	0 (0)
Dyspnea	18 (40.9)	16 (36.4)	2 (4.5)
Headache	16 (36.4)	16 (36.4)	0 (0)
Anorexia	16 (36.4)	16 (36.4)	0 (0)
Anemia	15 (34.1)	14 (31.8)	1 (2.3)
Alopecia	14 (31.8)	14 (31.8)	0 (0)
Arthralgia	14 (31.8)	14 (31.8)	0 (0)
Febrile neutropenia	3 (6.8)	1 (2.3)	2 (4.5)

*n* number

### Biomarkers

CTCs in blood were enumerated at baseline and during treatment to explore the correlation between CTC levels and response to EC. Of the 44 evaluable patients, 26 had baseline CTC data. Of these, 14 were CTC-positive (53.8%). There was no significant difference in the mean CTCs/7.5 mLs of blood at baseline between subtypes (t-test p = 0.6435). There was no significant association between CTC status at baseline and treatment response at first scan (Fisher p = 0.5901). 18 of the 26 patients had paired CTC data at baseline and follow-up, 2 of whom were CTC positive at baseline and turned CTC-negative on treatment. The change in CTC status was not significantly associated with response to treatment (Fisher p = 0.3355).

The median PFS was significantly shorter in patients who were CTC-positive at baseline compared to those who were CTC-negative (13.1 vs. 30.6 weeks, p = 0.011). Eighteen patients had on-treatment CTC data available (either during treatment or at the end of study). Of these, nine were CTC-positive (50%). The median PFS was significantly shorter in patients who were CTC-positive during treatment compared to those who were CTC-negative (13.1 weeks vs. 30.6 weeks, p = 0.035).

## Discussion

This trial was conducted to assess the safety, efficacy, and tolerability of EC for the treatment of patients with metastatic or ABC. CBR and PFS were 79.5% and 16.4 weeks respectively, comparing favorably to historic data of single agent eribulin for ABC (PFS 14.8 weeks) [14]. Our data demonstrate that EC has activity in extensively pretreated patients, as 68.2% of patients who enrolled had been treated with three to seven prior lines of chemotherapy. Of note, responses were observed in patients who had received prior anthracycline and taxane therapies.

Patients with both HR + and TNBC were enrolled. In particular, metastatic TNBC is an aggressive breast cancer subtype associated with poor clinical outcomes highlighting the importance of identifying novel treatment approaches. Previous phase III studies demonstrated statistically significant improvements in OS in patients with metastatic TNBC treated with eribulin versus treatment of physician choice in both subgroup and pooled analyses. Specifically, in a pooled analysis of the EMBRACE study and Study 301, eribulin significantly improved OS compared with TPC in patients with TNBC (HR: 0.74, p = 0.006) [16, 23]. In our study, 12 patients with metastatic TNBC received EC, with a CBR of 66.7% and a PR rate of 25%. The median PFS was 10.8 weeks with EC treatment. Among patients with metastatic TNBC, the heavily pre-treated patients had longer responses to EC than the less heavily-pretreated patients, although numbers are small; future larger studies can evaluate this further.

The adverse events of eribulin in this study is consistent with what has been reported in previous studies [14, 19]. In this study, the most frequently reported treatment related AEs were fatigue (68.2%), neutropenia (59.1%), nausea (56.8%), constipation (50%), peripheral neuropathy (47.7%), dyspnea (40.9%), headache (36.4%), and anorexia (36.4%), which reflect the known toxicity profiles of eribulin and cyclophosphamide. The most common grade 3/4 AE was neutropenia (47.7%; 6.8% febrile neutropenia). The incidence of neuropathy was 47.7%, but no patient experienced grade 3/4 neuropathy. Similarly, prior clinical trials also report high incidence of neutropenia and neuropathy with the use of eribulin. In the EMBRACE study, 52% of participants experienced neutropenia (grade 3: 8%, grade 4: 1%) and 35% of participants experienced peripheral neuropathy (grade 3: 8%, grade 4: 0.4%) [14]. In real-world studies of patients treated with eribulin grade 3/4 neutropenia occurred in 12% of patients and grade 3/4 neuropathy occurred in 2.6% of patients [24]. Overall, given that the patient population was heavily pre-treated in our study, it was reassuring that the toxicity profile of this combination chemotherapy regimen was similar to those previously reported in single agent studies.

In this study, we administered eribulin using a 21-day cycle at a dose of 1.4 mg/m^2^ on days 1 and 8 combined with cyclophosphamide at a fıxed dose of 600 mg/m^2^ on day 1 of each cycle. Previous studies demonstrated that eribulin was more tolerable when administered on a 21-day schedule compared to a 28-day schedule [25]. Alternative schedules of eribulin administration have been investigated to provide better tolerance in patients who experienced myelosuppression. A modified biweekly regimen which provides additional time for bone marrow recovery may potentially improve safety compared with the 21-day dosing regimen [26]. In a prospective phase 2 trial, biweekly eribulin (1.4 mg/m^2^ on days 1 and 15 of a 28-day cycle) was tolerable and had comparable antitumor activity in patients who were intolerant of the standard eribulin schedule [26]. Dose reductions due to neutropenia required patients to decrease to 500 mg/m^2^ cyclophosphamide (n = 17) and to 1.1 mg/m^2^ in eribulin (n = 12), consistent with expected hematologic toxicity in this heavily pre-treated population. Only five patients discontinued treatment due to AEs.

Previous studies have suggested that the presence of CTCs in patients with MBC is associated with a worse prognosis [27, 28] and can predict treatment response and progression [29]. Based on these prior work, we performed an exploratory CTC analysis to address whether CTC levels correlate with response to EC. Consistent with previous studies [27, 28], the CTC positivity rate was 53.8% (14 of 26 patients) at baseline. Median PFS was significantly shorter in patients who were CTC-positive at baseline or during treatment compared to those who were CTC-negative.

This study has several notable strengths. First, few studies have evaluated combination chemotherapy in patients with MBC who have received multiple lines of prior chemotherapy [14, 30]. Inclusion of this patient population in our study suggests that the EC regimen may be more generalizable to real-world treatment scenarios. Second, this trial included patients with multiple breast cancer subtypes: most patients had HR + /HER2- breast cancer, with a smaller number of patients with TNBC, and one patient with HR + /HER2 + disease. The RR to EC was higher in patients with HR + /HER2- disease compared to patients with TNBC, as expected, but our study was not powered to fully detect differences between subtypes. Third, the CTC data provides interesting correlative data, and we found that the median PFS was significantly shorter in patients who were CTC-positive at baseline compared to those who were CTC-negative, providing rationale to continue to study the prognostic and predictive value of CTCs in ABC.

This study also has several limitations. First, the primary endpoint of CBR provides important clinical information about response to EC, but this trial is not designed to provide data about OS. Second, since patients were heavily pre-treated, treatment history was fairly heterogenous, which clearly impacts both response to therapy and toxicity. This was a single-arm study so it is not possible to determine the efficacy of this regimen compared to others, and further studies are needed to clarify the efficacy of this regimen. Lastly, our study was conducted before regulatory approval of pembrolizumab plus chemotherapy for PD-L1 + metastatic TNBC, sacituzumab govitecan for metastatic TNBC and heavily pre-treated HR + /HER2- MBC, and trastuzumab deruxtecan for HER2-low MBC. However, as patients being treated with eribulin today will be even more heavily pre-treated than the patients in this trial, the activity of this combination regimen may have implications for current therapeutic options.

In conclusion, the results of this trial demonstrate that EC has antitumoral activity in heavily pretreated patients with locally ABC or MBC. Importantly, EC demonstrated a manageable tolerability profile. These results support the additional clinical development of EC as a novel treatment combination for the treatment of ABC.

### Supplementary Information

Below is the link to the electronic supplementary material.

Supplementary file1 (DOCX 30 KB)

## Data Availability

Enquiries about data availability should be directed to the authors.
