# Supplementary material for: A phase Ib/II study of eribulin in combination with cyclophosphamide in patients with advanced breast cancer
Source: Breast Cancer Res Treat. 2023 Oct 10;203(2):197–204. doi: 10.1007/s10549-023-07073-0 (PMC10787873; doi:10.1007/s10549-023-07073-0)
Supplement: Supplementary file 1 — Supplementary file1 (DOCX 30 KB) [file 10549_2023_7073_MOESM1_ESM.docx]

**Supplement 1:** Toxicity management guidelines for eribulin and cyclophosphamide

| **Toxicity** | **NCI-CTC grade, unless otherwise specified** | **Management of Eribulin dose** | **Eribulin dose upon resumption** | **Management of cyclophosphamide** | **Cyclophosphamide dose upon resumption** |
| --- | --- | --- | --- | --- | --- |
| Neutropenia (ANC <1000)  +/- Neutropenic fever | 1st episode | Delay dose* until afebrile and ANC ≥ 1000 | No change and add filgrastim | Delay dose until afebrile and ANC ≥ 1000 | No change and add filgrastim |
|  | 2nd episode | Delay dose until afebrile and ANC ≥ 1000 | No change | Delay dose until afebrile and ANC ≥ 1000 | Reduce to 500 mg/m2 and continue filgrastim |
|  | 3rd episode | Delay dose until afebrile and ANC ≥ 1000 | Reduce by one dose level | Delay dose until afebrile and ANC ≥ 1000 | Continue 500 mg/m2; continue filgrastim |
|  | 4th episode | Permanently discontinue | Permanently discontinue | Permanently discontinue | Permanently discontinue |
| Thrombocytopenia | Grade 2 or greater | Delay dose until resolved to ≥ Grade1 | No change | Delay dose until resolved to ≥ Grade1 | Reduce to 500 mg/m2 |
|  | Recurrent grade 2 or greater | Delay dose until resolved to ≥ Grade1 | Reduce by one dose level | Delay dose until resolved to ≥ Grade1 | No change |
|  | 3rd occurrence grade 2 or greater | Delay dose until resolved to ≥ Grade1 | No change | Delay dose until resolved to ≥ Grade1 | Reduce to 400 mg/m2 |
|  | 4th occurrence grade 2 or greater | Permanently discontinue | Permanently discontinue | Permanently discontinue | Permanently discontinue |
| Rash | 1 or 2 | Continue treatment; supportive care | No change** | Continue treatment | No change |
|  | 3 | Delay dose until resolved to ≥ Grade1; supportive care | No change** | Delay dose until resolved to ≥ Grade1 | No change |
|  | Recurrent grade 3 | Delay dose until resolved to ≥ Grade1; supportive care | Reduce by one dose level | Delay dose until resolved to ≥ Grade1 | No change |
|  | 3rd occurrence  Grade 3 | Permanently discontinue; supportive care | Permanently discontinue | Permanently discontinue | Permanently discontinue |
| GI toxicity: diarrhea, nausea, vomiting | 1, 2 | Continue treatment | No change; maximize supportive  treatment | No change; maximize supportive treatment | No change; maximize supportive  treatment |
|  | 3 | Delay dose until resolved to ≥ Grade1 | No change | Delay dose until resolved to ≥ Grade1 | No change |
|  | Recurrent 3 | Delay dose until resolved to ≥ Grade1 | No change | Delay dose until resolved to ≥ Grade1 | Reduce to 500 mg/  m^2^ |
|  | 3rd occurrence | Delay dose until resolved to ≥ Grade1 | Reduce by one dose level | Delay dose until resolved to ≥ Grade1 | No change |
|  | 4th occurrence grade 3 or 4 | Permanently discontinue | Permanently discontinue | Permanently discontinue | Permanently discontinue |
| Liver function abnormalities  (ALT, AST, ALP only) | 1, 2 | Continue treatment | No change | Continue treatment | No change |
|  | 3 and > 2x baseline | Delay dose until resolved to < Grade 3 | No change | Delay dose until resolved to < Grade 3 | Reduce to 500 mg/  m^2^ |
|  | recurrent 3 and > 2x baseline | Delay dose until resolved to < Grade 3 | reduce by one dose level | Delay dose until resolved to < Grade 3 | No change |
|  | 4th occurrence grade 3 or 4 | Permanently discontinue | Permanently discontinue | Permanently discontinue | Permanently discontinue |
| Neuropathy (motor and  sensory) | 1, 2 | Continue treatment | No change | continue treatment | No change |
|  | 3 | Delay dose until resolved to < Grade 3 | No change | Delay dose until resolved to < Grade 3 | No change |
|  | Recurrent 3 | Delay dose until resolved to < Grade 3 | Reduce by one dose level | Delay dose until resolved to < Grade 3 | No change |
|  | 3rd occurrence 3 | Permanently discontinue | Permanently discontinue | Permanently discontinue | Permanently discontinue |

Abbreviations: ALT, alanine aminotransferase; ALP, alkaline phosphatase; ANC, absolute neutrophil count; AST, aspartate aminotransferase

* A cycle is a 3-week course. If treatment needs to be held for >48 hours, the dose should be held for that week and resumed the following week. If doses are held for more than 3 weeks, the patients must be removed from study.

** If Grade 2 rash develops but resolves to grade ≤ 1 within 7 days allowing the patient to resume treatment, the patient should be restarted at the same dose.
